# Subcomponents and Connectivity of the Inferior Fronto-Occipital Fasciculus Revealed by Diffusion Spectrum Imaging Fiber Tracking

**DOI:** 10.3389/fnana.2016.00088

**Published:** 2016-09-23

**Authors:** Yupeng Wu, Dandan Sun, Yong Wang, Yibao Wang

**Affiliations:** ^1^Department of Neurosurgery, The First Affiliated Hospital of China Medical UniversityShenyang, China; ^2^Department of Cardiovascular Ultrasound, The First Affiliated Hospital of China Medical UniversityShenyang, China

**Keywords:** inferior fronto-occipital fasciculus, diffusion spectrum imaging, asymmetry, language, tractography

## Abstract

The definitive structure and functional role of the inferior fronto-occipital fasciculus (IFOF) are still controversial. In this study, we aimed to investigate the connectivity, asymmetry, and segmentation patterns of this bundle. High angular diffusion spectrum imaging (DSI) analysis was performed on 10 healthy adults and a 90-subject DSI template (NTU-90 Atlas). In addition, a new tractography approach based on the anatomic subregions and two regions of interest (ROI) was evaluated for the fiber reconstructions. More widespread anterior-posterior connections than previous “standard” definition of the IFOF were found. This distinct pathway demonstrated a greater inter-subjects connective variability with a maximum of 40% overlap in its central part. The statistical results revealed no asymmetry between the left and right hemispheres and no significant differences existed in distributions of the IFOF according to sex. In addition, five subcomponents within the IFOF were identified according to the frontal areas of originations. As the subcomponents passed through the anterior floor of the external capsule, the fibers radiated to the posterior terminations. The most common connection patterns of the subcomponents were as follows: IFOF-I, from frontal polar cortex to occipital pole, inferior occipital lobe, middle occipital lobe, superior occipital lobe, and pericalcarine; IFOF-II, from orbito-frontal cortex to occipital pole, inferior occipital lobe, middle occipital lobe, superior occipital lobe, and pericalcarine; IFOF-III, from inferior frontal gyrus to inferior occipital lobe, middle occipital lobe, superior occipital lobe, occipital pole, and pericalcarine; IFOF-IV, from middle frontal gyrus to occipital pole, and inferior occipital lobe; IFOF-V, from superior frontal gyrus to occipital pole, inferior occipital lobe, and middle occipital lobe. Our work demonstrates the feasibility of high resolution diffusion tensor tractography with sufficient sensitivity to elucidate more anatomical details of the IFOF. And we provides a new framework for subdividing the IFOF for better understanding its functional role in the human brain.

## Introduction

The inferior fronto-occipital fasciculus (IFOF) is one of the first major association fiber systems to be recognized and depicted in the human brain (Schmahmann and Pandya, 2007). As a long associative bundle, the IFOF passes through in the depth of temporal lobe and insula, connecting occipital cortex, temporo-basal areas, and superior parietal lobe to the frontal lobe (Martino et al., 2010). Also, there are some natural crossings of the IFOF with other tracts, including the superior longitudinal fasciculus (SLF), arcuate fascicle (AF), inferior longitudinal fasciculus (ILF), and middle longitudinal fascicle (MdLF). Several studies suggested that the IFOF possibly played an important role in reading, attention, and visual processing (Catani and Thiebaut de Schotten, 2008).

Traditionally, modern autoradiography in monkeys are considered as the “gold standard” for guiding human white matter connective patterns, however, it has been demonstrated that there is not existence of a human equivalent of the IFOF in the monkey brain (Schmahmann et al., 2007; Forkel et al., 2014). As for this, post-mortem dissection and diffusion tensor imaging (DTI) technique have been widely adopted to explore the anatomical structures of the IFOF. In 2013, Sarubbo et al. investigated the frontal terminations of the IFOF on 10 hemispheres using fiber dissection technique, and proposed that this fiber bundle could be segmented into different subcomponents for future functional analysis (Sarubbo et al., 2013). Two possible layers of the IFOF were identified by Sarubbo et al. according to the fiber frontal terminations. The first layer was superficial and antero-superiorly directed, associating with the inferior frontal gyrus. The second one was deeper and composed of three discrete fan-shaped portions: Anterior, middle, and posterior. The anterior component was connected with the orbito-frontal cortex and frontal pole. The middle component was directed to the lateral orbito-frontal cortex and middle frontal gyrus. The posterior one terminated in the dorso-lateral prefrontal cortex and middle frontal gyrus (Sarubbo et al., 2013). Coincident results were found by De Benedictis et al. (2012). However, neither of them mentioned the connections of the subcomponents to the posterior terminations. In another post-mortem dissection study, Martino et al. firstly reported new cortical terminations (i.e., postero-basal temporal portions and superior parietal lobe) except as the occipital cortex. And they suggested that there were two different subcomponents in the IFOF. One was a dorsal and superficial subpart that connected the superior parietal lobe and the posterior part of the superior and middle occipital cortex with the frontal lobe. The other was a ventral and deep subpart that connected the posterior part of the inferior occipital cortex and the posterior temporo-basal portions with the frontal lobe. But, they failed to identify their cortical terminations within the frontal lobe (Martino et al., 2010). The controversial results between studies might be attributed to the limitations of fiber dissection methods. First, this technique depends on the quality of the specimens, especially difficult to be performed in brains of elderly subjects with lacunar infarcts (Martino et al., 2011). Second, the dissection is limited to be applied in different crossing fiber termination regions.

As an alternative method, DTI technique provides a unique opportunity to study white matter architecture *in vivo* as 3D space. For the last decades, it has strongly contributed to our knowledge about fiber pathways in the human brain (Catani et al., 2002). Still, the detailed anatomical definition of the IFOF exists some controversies within reported DTI studies. For instance, some researchers described that the IFOF connected the fronto-marginal gyrus and lateral orbito-frontal gyrus with the lingual gyrus, inferior occipital gyrus, and inferior part of the middle occipital gyrus (Lawes et al., 2008). Whereas, others considered that the posterior terminations of the IFOF was at the level of the middle and posterior temporal lobe (Fernandez-Miranda et al., 2008a). Recently, Thiebaut de Schotten et al. reported that it probably had a contribution from the medial parietal lobe (Thiebaut de Schotten et al., 2012). We speculate that the technique DTI limitations may impede accurate depiction of the IFOF, such as the inability to map fiber ends of the white matter before contacting the cortical mantle, failure to solve complicated fiber crossings and to follow bundles within fiber tracts, and excessive false fiber continuity generating pseudotracts (Fernandez-Miranda, 2013). In order to resolve the complex fiber crossings and partial-volume effect that typically affect DTI tractography data, tractography algorithms based on diffusion spectrum imaging (DSI) reconstructed by generalized q-sampling imaging (GQI) as a high-angular-resolution diffusion imaging (HARDI) method have been developed (Wedeen et al., 2005; Yeh et al., 2010). This approach leverages high directional sampling of diffusion imaging space to get better resolution of underlying white matter geometry for tractography. Our previous studies have provided accurate replication of complex known neuroanatomical features where DTI failed (Wang et al., 2013; Fernandez-Miranda et al., 2014; Leng et al., 2016; Wu et al., 2016).

In this study, we aimed to investigate the connectivity, asymmetry and segmentation of the left and right IFOF in a subject-specific approach (10 subjects) and a template approach (NTU-90 Atlas) using HARDI tractography. In addition, we evaluated a new tractography approach for the fiber tracking of IFOF based on the anatomic subregions and two regions of interest (ROI). We complemented this analysis compared with recent post-mortem dissection and functional neuroimaging studies of the IFOF to confirm the underlying neuroanatomy. Our study provides a more accurate and detailed description of the connectional architecture of the subcomponents within the IFOF, allowing a comprehensive morphological basis for further functional studies in the human brain.

## Materials and methods

### Participants

Ten volunteers (3 males, 7 females; all right handed; age range: 23–40 years) were included in the present study. No history of neurological and psychiatric disorders was present in study sample. All participants gave informed written consent prior to testing. The procedures used here were approved by the institutional review board, including the ethics committee at China Medical University.

Except as subject-specific analysis, we also conducted fiber tracking on a publicly available DSI template of 90 subjects (NTU-90 atlas). It includes 45 males and 45 female subjects. The age of the 90 subjects ranges from 18 to 60 years, and the mean ages of the male and female subjects are 32.58 and 33.58 years. The spatial resolution of the template is 2 mm (Yeh and Tseng, 2011). The NTU-90 template is freely downloadable at http://dsi-studio.labsolver.org.

### Image acquisition and reconstruction

All studies were performed on a 3-T Tim Trio System (Siemens) with a 32-channel head coil to acquire DSI data. A head stabilizer was used to prevented head motion. This involved a 43-min, 257-direction scan using a twice-refocused spin-echo echo-planar imaging sequence and multiple *q*-values [echo time (TE) = 157 ms, repetition time (TR) = 9.916 ms, field of view (FoV) = 231 × 231 mm, voxel size = 2.4 × 2.4 × 2.4 mm, b max = 7000 s/mm^2^] (Wedeen et al., 2005). We also included the high-resolution anatomical imaging to be as the anatomical comparisons, employing a 9-min T1-weighted axial magnetization prepared rapid gradient echo (MPRAGE) sequence (TE = 2.63 ms, TR = 2110 ms, flip angle = 8°, 176 slices, voxel size = 0.5 × 0.5 × 1.0 mm^3^, FoV = 256 × 256 mm^2^). DSI data was reconstructed with a generalized Q-sampling imaging (GQI) approach (Yeh et al., 2010). The orientation distribution functions (ODFs) were reconstructed to 362 discrete sampling directions and a mean diffusion distance of 1.2 mm.

### Fiber tracking and analysis

The DSI Studio software was used for the fiber-tracking performing a multiple fiber version of the streamline tracking algorithm. The DSI Studio software is an open-source diffusion MRI analysis tool, which is freely downloadable at http://dsi-studio.labsolver.org. The fiber tracts were generated by whole brain seeding, and the tracts passing the ROI were implemented. In voxels with multiple fiber orientations, fiber tracking was initiated separately for each orientation, and fiber progression continued with a step size of 1.0 mm, minimum fiber length of 20 mm, and turning angle threshold of 60°. If multiple fiber orientations existed in the current progression location, the fiber orientation that was nearest to the incoming direction and formed a turning angle smaller than 60° was selected to determine the next moving direction (Fernandez-Miranda et al., 2014). To smooth each track, the next moving directional estimate of each voxel was weighted by 20% of the previous incoming direction and 80% of the nearest fiber orientation. This progression was repeated until the quantitative anisotropy (QA) of the fiber orientation dropped below a preset threshold (0.03–0.06 depending on the subject) or there was no fiber selected within the 60° angular range in the progression (Yeh et al., 2013). Once tracked, all streamlines were saved in the TrackVis file format.

**Figure 1:**
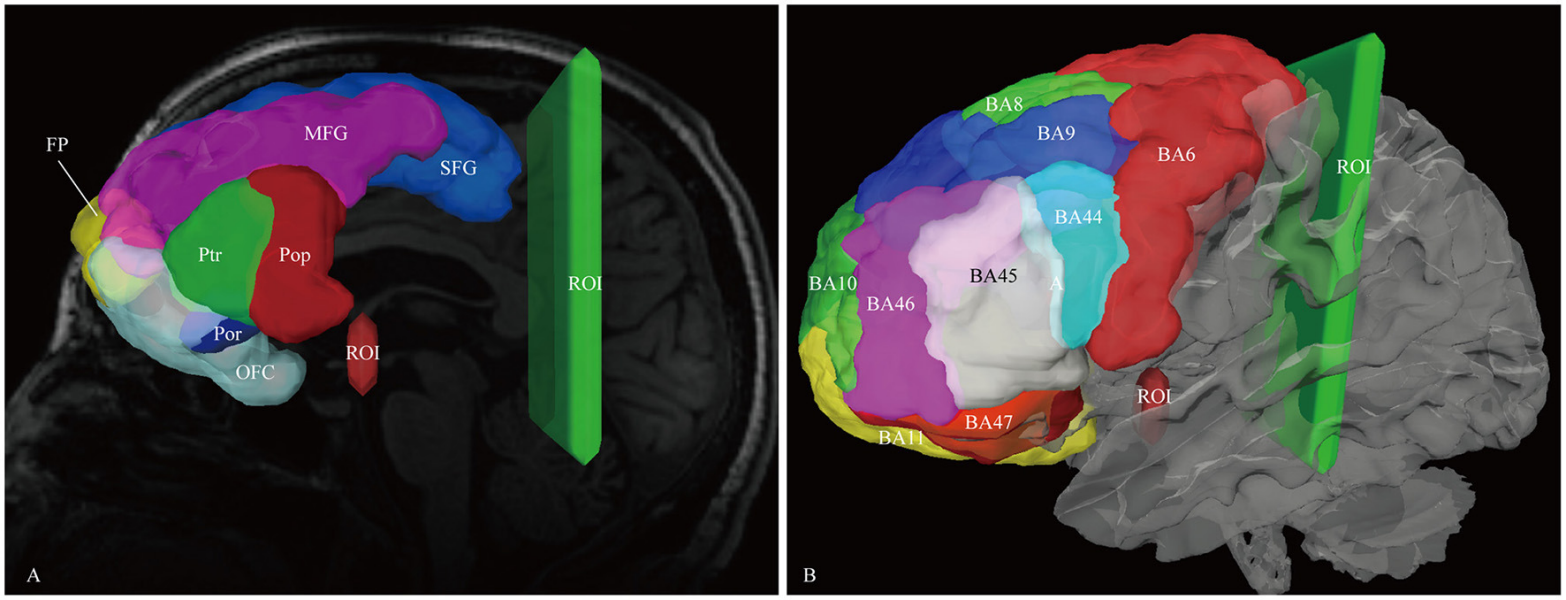
*****In vivo*** fiber tractography of the IFOF. (A)** Frontal lobe labels were used as the seeding regions for the reconstruction of the IFOF, including the superior frontal gyrus (SFG, blue), middle frontal gyrus (MFG, pink), inferior frontal gyrus [i.e., pars opercularis (Pop, red), pars triangularis (Ptr, green), and pars orbitalis (Por, blue)], frontal polar (FP, yellow) and orbito-frontal cortex (OFC, cyan). Two ROI masks were drawn to select only the fibers that passed from the frontal lobe to the posterior region. One was around the ventral part of the external capsule on the coronal quantitative anisotropy color map, the other was on the coronal plane at the level of the central sulcus. **(B)** Brodmann areas (BA) overlaid on the white matter surface. ROI, region of interest.

For reconstruction of the IFOF, we assumed that the IFOF fibers only originated from the frontal lobe. The bilaterally frontal lobe cortical ROIs were all employed acting as the seeding regions, including the superior frontal gyrus, middle frontal gyrus, inferior frontal gyrus (i.e., pars opercularis, pars triangularis, and pars orbitalis), frontomarginal gyrus, transverse fronto-polar gyrus, and orbito-frontal cortex(i.e., lateral and medial orbito-frontal cortex). Two ROI masks were drawn to select only the fibers that passed from the frontal lobe to the posterior region. One was around the ventral part of the external capsule on the coronal QA color map, the other was on the coronal plane at the level of the central sulcus (Figure 1). In addition, for analyzing the spatial relationship of the IFOF with adjacent association tracts, we performed fiber reconstruction of the inferior longitudinal fascicles (ILF), optic radiation (OR), uncinate fasciculus (UF) and middle longitudinal fascicle (MdLF). For the ILF, two different ROIs were used: One along the anterior temporal region and another at the occipital region (Fernandez-Miranda et al., 2008a). For the OR, two different ROIs were applied: One at the thalamus region and another at the occipital region (Kamali et al., 2014). For the UF, the superior temporal gyrus, middle temporal gyrus, and temporal pole were performed as the seed regions (Leng et al., 2016). For the MdLF, the superior temporal gyrus was served as the seed region. The ROI mask was used on the coronal plane along the precentral region (Wang et al., 2013).

### Cytoarchitectonic and anatomic segmentation

Brodmann areas and anatomical segmentation were both used to perform the cortical parcellation. DSI Studio uses linear transformation to register Talairach atlas on subject's diffusion space. The Talairach coordinate system provides anatomical and functional information (Nowinski, 2005). For comparison, FreeSurfer (http://surfer.nmr.mgh.harvard.edu) was used to segment cortical gyral ROIs based on previous brain atlases using each participant's T_1_-weighted magnetization prepared rapid axial gradient echo (MPRAGE) image (Desikan et al., 2006). For the template, ICBM-152 T_1_ template image was performed in FreeSurfer (Fonov et al., 2011).

### Statistical analysis

Statistical analyses were carried out using SPSS 16.0 (SPSS, Chicago, IL). For quantifying and comparing the right and left IFOF as well as the contribution of each seeding region, we calculated the volume of streamlines based on counting the number of voxels occupied by the fiber trajectories (streamlines). Continuous variables were presented as mean ± standard deviation. *T*-test was used to determine variances between the tract volume of left hemispheres and that of right hemispheres. All originating and terminating distributions of the five subcomponents between males and females were evaluated by chi-square (χ*2*) test. *P* ≤ 0.05 was considered to be statistically significant.

## Results

### The trajectory of the IFOF in “subject-specific” tractography approach

**Figure 2:**
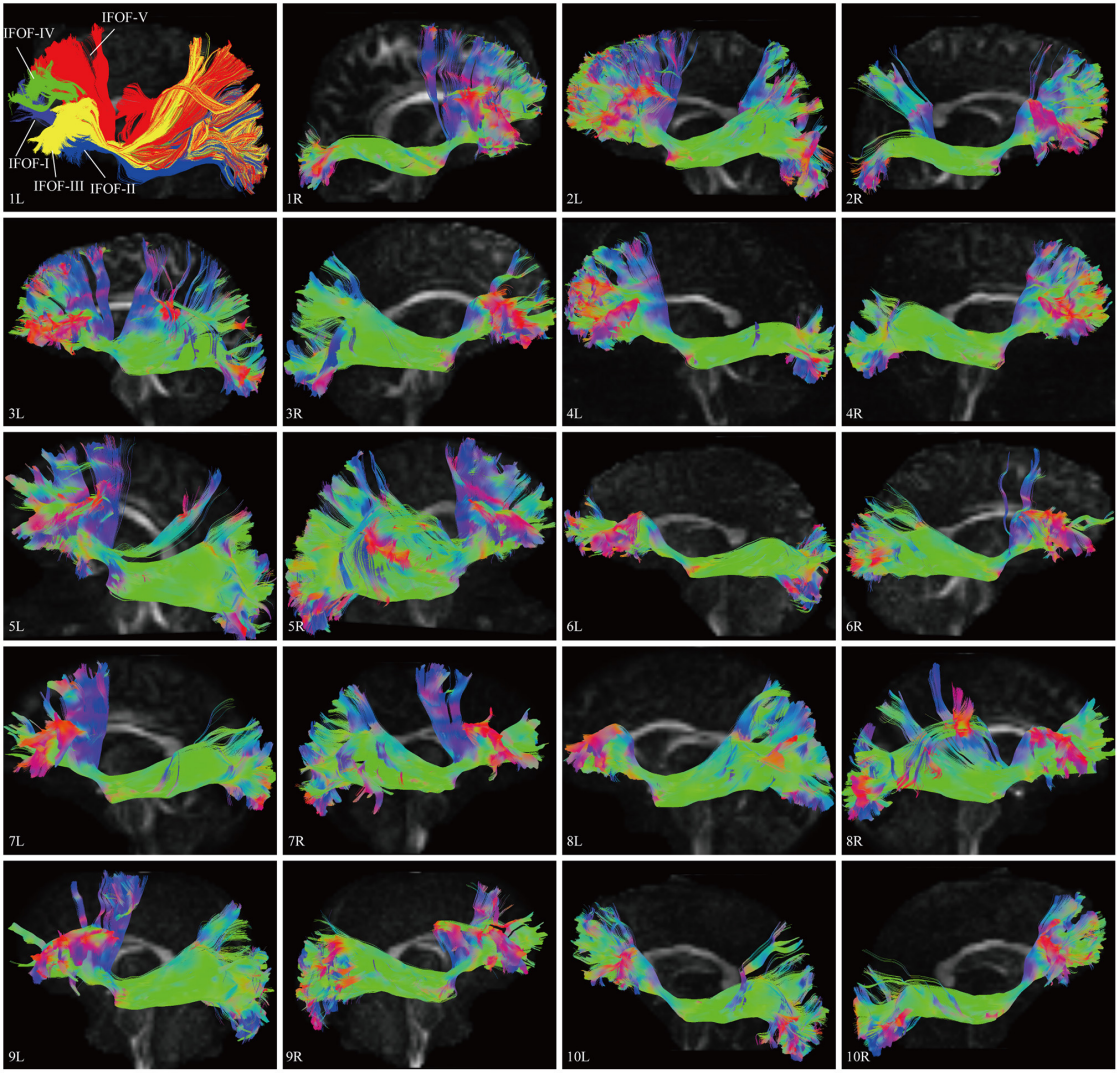
**DSI tractography studies of the IFOF in 20 hemispheres of 10 subjects on sagittal view**. Order: Subject 1, 2, 3, 4, 5, 6, 7, 8, 9, 10. The IFOF was noted as a long-ranged and bowtie-shaped pathway at the inferior base of the cerebrum in all 20 hemispheres. As the fibers leaved the originations, they narrowed as a compact fascicle at the level of the external/extreme capsules, then fanned out to the terminations. Greater inter-subjects connective variabilities were demonstrated with a maximum of 40% overlap in the central part. L, left; R, right.

In this study, the fiber tractography study consistently noted a large bundle of fibers projecting from the frontal seed regions toward the posterior terminations in all 20 hemispheres (Figure 2). Importantly, this distinct pathway demonstrated a greater inter-subjects connective variability with a maximum of 40% overlap in its central part. This group of fibers mainly originated from the orbito-frontal cortex (lateral and medial orbito-frontal cortex), frontal polar cortex (i.e., fronto-marginal gyrus and transverse fronto-polar gyrus), superior frontal gyrus and inferior frontal gyrus (i.e., pars opercularis, pars triangularis, pars orbitalis), some fibers also came from the middle frontal gyrus (12 out of 20 hemispheres). As the fibers leaved the originations, they narrowed as a compact fascicle at the level of the external/extreme capsules. Then, the fibers continued posteriorly through the temporal lobe. Subsequently, they fanned out before radiating to the terminations. In the sagittal view, it can be clearly noticed that the IFOF was a long-ranged and bowtie-shaped pathway at the inferior base of the cerebrum. The posterior terminations of the IFOF mainly included the pericalcarine (cuneus and lingual gyrus), fusiform gyrus, occipital pole, inferior occipital lobe, middle occipital lobe, and superior occipital lobe. Some fibers also terminated in the superior parietal lobe (10 out of 20 hemispheres), angular gyrus (10 out of 20 hemispheres) and postcentral gyrus (5 out of 20 hemispheres), and with a minor connection probably in the inferior temporal gyrus (1 out of 20 hemispheres) and middle temporal gyrus (1 out of 20 hemispheres) (Table 1).

**Table 1:** **Cortical connections of the five segments of the IFOF**.

**No**	**Side**	**IFOF-I connection**		**IFOF-II connection**		**IFOF-III connection**		**IFOF-IV connection**		**IFOF-V connection**	
		**Anterior**	**Posterior**	**Anterior**	**Posterior**	**Anterior**	**Posterior**	**Anterior**	**Posterior**	**Anterior**	**Posterior**
1	L	FP	IOL,MOL,SOL,SPL,Opo	OFC	IOL,MOL,SOL,Opo,SPL,Pca,FG	Pop,Ptr,Por	PG,IOL,MOL,SOL,Opo,AG,SPL	MFG	PG,IOL,MOL,SOL,Opo,AG,SPL	SFG	PG,IOL,MOL,SOL,Opo,AG,SPL
	R	FP	IOL,Opo	OFC	IOL,MOL,Opo,Pca,FG	Pop,Ptr,Por	IOL,MOL,Opo	–		SFG	IOL,MOL,Opo
2	L	FP	IOL,SOL,SPL,Opo,Pre,Pca	OFC	IOL,SOL,SPL,Opo,Pre,Pca	Pop,Ptr,Por	AG,IOL,MOL,Opo	MFG	AG,IOL,MOL,Opo,Pca	SFG	AG,IOL,MOL,SPL,Opo,Pca
	R	FP	IOL,Opo,SPL	OFC	IOL,Opo,SPL	Ptr,Por	IOL,Opo,Pca	MFG	IOL,Opo	SFG	IOL,Opo,SPL,Pca
3	L	FP	IOL,MOL,SOL,Opo,Pca	OFC	IOL,MOL,SOL,Opo,Pca	Ptr,Por	PG,AG,IOL,MOL,SOL,Opo,FG	MFG	IOL,MOL,Opo,Pca,FG	SFG	IOL,MOL,Opo,Pca,FG
	R	FP	IOL,MOL,SOL,Opo,SPL,Pca,FG	OFC	IOL,MOL,SOL,Opo,Pca,FG	Ptr,Por	AG,IOL,MOL,SOL,Opo,SPL,Pca,FG	MFG	IOL,Opo,Pca,FG	SFG	IOL,Opo
4	L	FP	IOL,MOL,Opo,FG	OFC	IOL,MOL,Opo,Pca	Pop,Ptr,Por	IOL,MOL,SOL,Opo,Pca	MFG	IOL,MOL,SOL,Opo,Pca	SFG	IOL,MOL,SOL,Opo,Pca
	R	FP	IOL,MOL,SOL,Opo,Pca,FG	OFC	IOL,MOL,SOL,Opo,Pca,FG	Ptr,Por	IOL,MOL,SOL,Opo,Pca	MFG	MOL,SOL,Opo,Pca	SFG	MOL,SOL,Opo,Pca
5	L	FP	IOL,Opo,Pca,FG	OFC	IOL,MOL,SOL,SPL,Opo,Pca,FG	Pop,Ptr,Por	PG,IOL,MOL,SOL,SPL,Opo,Pca,FG	MFG	IOL,MOL,SPL,Opo,Pca	SFG	PG,IOL,MOL,SOL,Opo,AG,SPL,Pca
	R	FP	PG,IOL,MOL,SOL,Opo,SPL,Pca	OFC	AG,IOL,MOL,SOL,Opo,SPL,Pca	Ptr,Por	PG,AG,IOL,MOL,SOL,Opo,SPL,Pca	–		SFG	SOL,SPL
6	L	FP	IOL,MOL,Opo,Pca	OFC	IOL,MOL,Opo,Pca	Ptr,Por	IOL,MOL,Opo	–		–	
	R	FP	IOL,MOL,Opo,Pca,FG	OFC	Opo,Pca,FG	Pop,Ptr,Por	IOL,MOL,Opo,Pca,FG	–		SFG	Opo,MOL
7	L	FP	IOL,MOL,SOL,SPL,Opo,Pca	OFC	IOL,MOL,Opo,Pca	Pop,Ptr,Por	IOL,MOL,Opo,Pca	MFG	IOL,MOL,Opo,Pca	SFG	IOL,MOL,Opo,Pca
	R	FP	IOL,MOL,SOL,Opo,Pca,FG	OFC	IOL,MOL,SOL,Opo,Pca	Ptr,Por	AG,IOL,MOL,SOL,SPL,Pca,ITG	MFG	IOL,MOL,Opo,Pca	SFG	SOL,ITG
8	L	FP	AG,IOL,MOL,SOL,SPL	OFC	AG,IOL,MOL,SOL,SPL	Ptr,Por	AG,IOL,MOL,SOL,SPL	–		–	
	R	FP	IOL,Opo,Pca,FG	OFC	IOL,Opo,FG,Pca	Pop,Ptr,Por	PG,MTG,IOL,MOL,SOL,SPL,Opo.FG	–		SFG	IOL,Opo,FG
9	L	FP	MOL,SPL,Opo	OFC	AG,SOL,SPL,Opo,Pca	Pop,Ptr,Por	AG,SOL,SPL,Opo,Pca	–		SFG	SOL,SPL,Opo
	R	FP	IOL,MOL,SOL„Opo,Pca	OFC	AG,IOL,MOL,SOL,SPL,Opo,Pca	Pop,Ptr,Por	AG,IOL,MOL,SOL,Opo,Pca	–		SFG	Opo
10	L	FP	IOL,MOL,SOL,Opo,Pca	OFC	IOL,MOL,SOL,Opo,FG,Pca	Ptr,Por	IOL,MOL,SOL,Opo	MFG	IOL,MOL,SOL,Opo,FG	SFG	IOL,MOL,SOL,FG,Opo
	R	FP	IOL,MOL,Opo,FG,Pca	OFC	IOL,MOL,Opo,FG,Pca	Pop,Ptr,Por	IOL,MOL,Opo,FG,Pca	MFG	Opo,Pca	SFG	IOL,Opo,FG,Pca
NTU-90	L	FP	IOL,LG	OFC	IOL,MOL,LG	Ptr,Por	PG,IOL,MOL,SOL,SPL,LG	MFG	IOL,MOL,SOL,SPL	SFG	PG,IOL,MOL,SOL,SPL,FG,Supa
	R	FP	LG	OFC	IOL,LG	Pop,Ptr,Por	AG,STG,MTG,IOL,MOL,SOL,SPL,LG	MFG	IOL,MOL,SOL,SPL	SFG	AG,STG,MTG,PG,IOL,MOL,SOL,SPL,FG

*SFG, superior frontal gyrus; MFG, middle frontal gyrus; Pop, pars opercularis; Ptr, pars triangularis; Por, pars orbitalis; FP, frontal polar cortex; OFC, orbito-frontal cortex; STG, superior temporal gyrus; MTG, middle temporal gyrus; ITG, inferior temporal gyrus; FG, fusiform gyrus; LG, lingual gygus; PG, postcentral gygus; IOL, inferior occipital gyrus; AG, angular gyrus; SOL, superior occipital lobe; MOL, middle occipital lobe; SPL, superior parietal lobe; Supra, supramarginal gyrus; Opo, occipital pole; Pca, pericalcarine; Pre, precuneus; L, left hemisphere; R, right hemisphere*.

### Segmentation and brain connectivity of the IFOF

In order to find common patterns of connectivity within the IFOF, we investigated the structural interconnectivity between frontal lobe and posterior regions. Anatomical comparisons between the anterior and posterior cortex regions in the human brain, the frontal lobe is more easily recognizable for the fiber tracking. As for this, five different cortical areas of originations of the IFOF fibers in the frontal region were identified: Frontal polar cortex (i.e., fronto-marginal gyrus and transverse fronto-polar gyrus), orbito-frontal cortex (i.e., lateral and medial orbito-frontal cortex), inferior frontal gyrus (i.e., pars opercularis, pars triangularis and pars orbitalis), middle frontal gyrus, and superior frontal gyrus. Based on the frontal anatomic landmarks, five potential subcomponents of the IFOF connecting the different cortical and subcortical regions were visualized. IFOF-I originated from the frontal polar cortex, via the external/extreme capsules, then connected with the inferior occipital lobe [19/20, (19 out of 20 hemispheres)], the occipital pole (19/20), the middle occipital lobe (15/20), the pericalcarine (14/20), the superior occipital lobe (11/20), the superior parietal lobe (8/20), the fusiform gyrus (8/20), the postcentral gygus (1/20), and the precuneus (1/20). IFOF-II originated from the orbito-frontal cortex, via the external/extreme capsules, then connected with the occipital pole (19/20), the inferior occipital gyrus (18/20), the pericalcarine (18/20), the middle occipital lobe (15/20), the superior occipital lobe (12/20), the fusiform gyrus (9/20), the superior parietal lobe (8/20), the angular gyrus (4/20), and the precuneus (1/20). IFOF-III originated from the inferior frontal gyrus, via the external/extreme capsules, then connected with the inferior occipital gyrus (19/20), the middle occipital lobe (18/20), the occipital pole (18/20), the superior occipital lobe (13/20), the pericalcarine (12/20), the angular gyrus (9/20), the superior parietal lobe (8/20), the fusiform gyrus (6 /20), the postcentral gygus (5/20), the inferior temporal gyrus (1/20), and the middle temporal gyrus (1/20). IFOF-IV originated from the middle frontal gyrus, via the external/extreme capsules, then connected with the occipital pole (12/20), the inferior occipital gyrus (10/20), the middle occipital lobe (9/20), the pericalcarine (9/20), the superior occipital lobe (4/20), the angular gyrus (2/20), the superior parietal lobe (2/20), the postcentral gygus (1/20), and the fusiform gyrus (1/20). Finally, IFOF-V originated from the superior frontal gyrus, via the external/extreme capsules, then connected with the occipital pole (16/20), the inferior occipital gyrus (12/20), the middle occipital lobe (10/20), the superior occipital lobe (8/20), the pericalcarine (8/20), the superior parietal lobe (6/20), the fusiform gyrus (4/20), the angular gyrus (3/20), the postcentral gygus (2/20), and the inferior temporal gyrus (1/20) (Table 1).

As the subcomponents passed through the anterior floor of the external capsule, the fibers were mixed together and radiated to the posterior terminations (Figure 4). In fact, it was impossible for us to differentiate the subcomponents if we portrayed the fiber course from the posterior connections. Even so, two characteristic connections in the subcomponents were found. First, the IFOF-I was largely located on the bottom to the others, while the IFOF-V was slightly more superior than the others, despite their overlaps. Second, the fibers terminating in the angular gyrus was mainly come from the IFOF-III (Figure 4A).

### “Template” tractography approach

**Figure 3:**
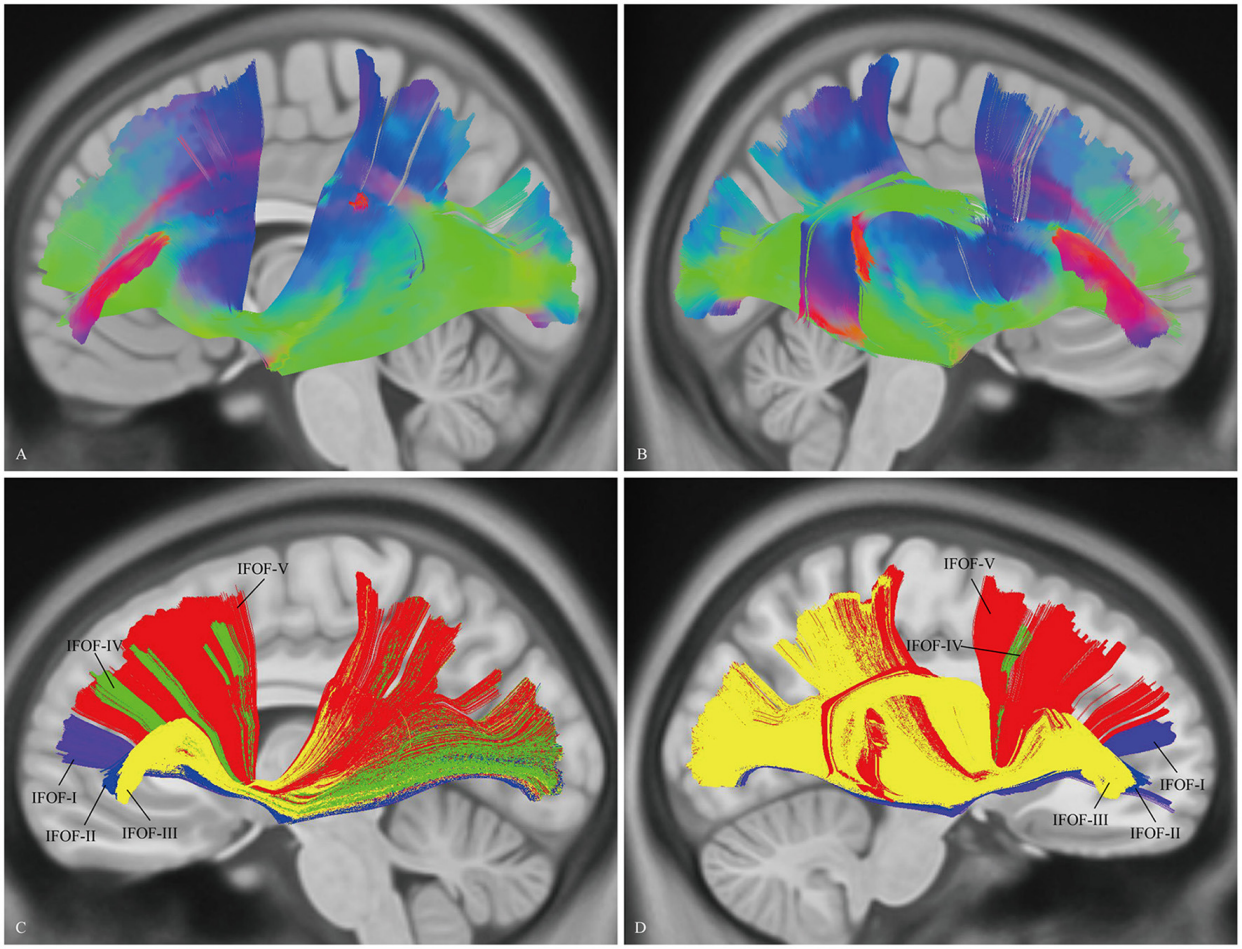
*****In vivo*** fiber tractography of the IFOF on the DSI template**. **(A,B)** The NTU-90 showed an extended pattern compared with the subject-specific results. **(C,D)** A consistent pattern of five segmentations was confirmed within both hemispheres of the template. L, left; R, right.

The DSI template (NTU-90) showed an extended pattern compared with the subject-specific results, similarly to the analysis of the subject 1L and the subject 5R. Moreover, a consistent pattern of five segmentations was confirmed within both hemispheres of the NTU-90 template (Figure 3). Briefly, IFOF-I connected the frontal polar cortex with the inferior occipital gyrus and the lingual gygus. IFOF-II connected the orbito-frontal cortex with the inferior occipital gyrus, the middle occipital lobe, and the lingual gygus. IFOF-III connected the inferior frontal gyrus with the inferior occipital gyrus, the middle occipital lobe, the superior occipital lobe (13/20), the pericalcarine, the angular gyrus, the superior parietal lobe, the superior temporal gyrus, the middle temporal gyrus, and the lingual gygus. IFOF-IV connected the middle frontal gyrus with the inferior occipital gyrus, the middle occipital lobe, the superior occipital lobe, and the superior parietal lobe. IFOF-V connected the superior frontal gyrus with the postcentral gygus, the inferior occipital lobe, the middle occipital lobe, the superior occipital lobe, the superior parietal lobe, the fusiform gyrus, the supramarginal gyrus, the angular gyrus, the superior temporal gyrus, and the postcentral gygus (Table 1).

**Figure 4:**
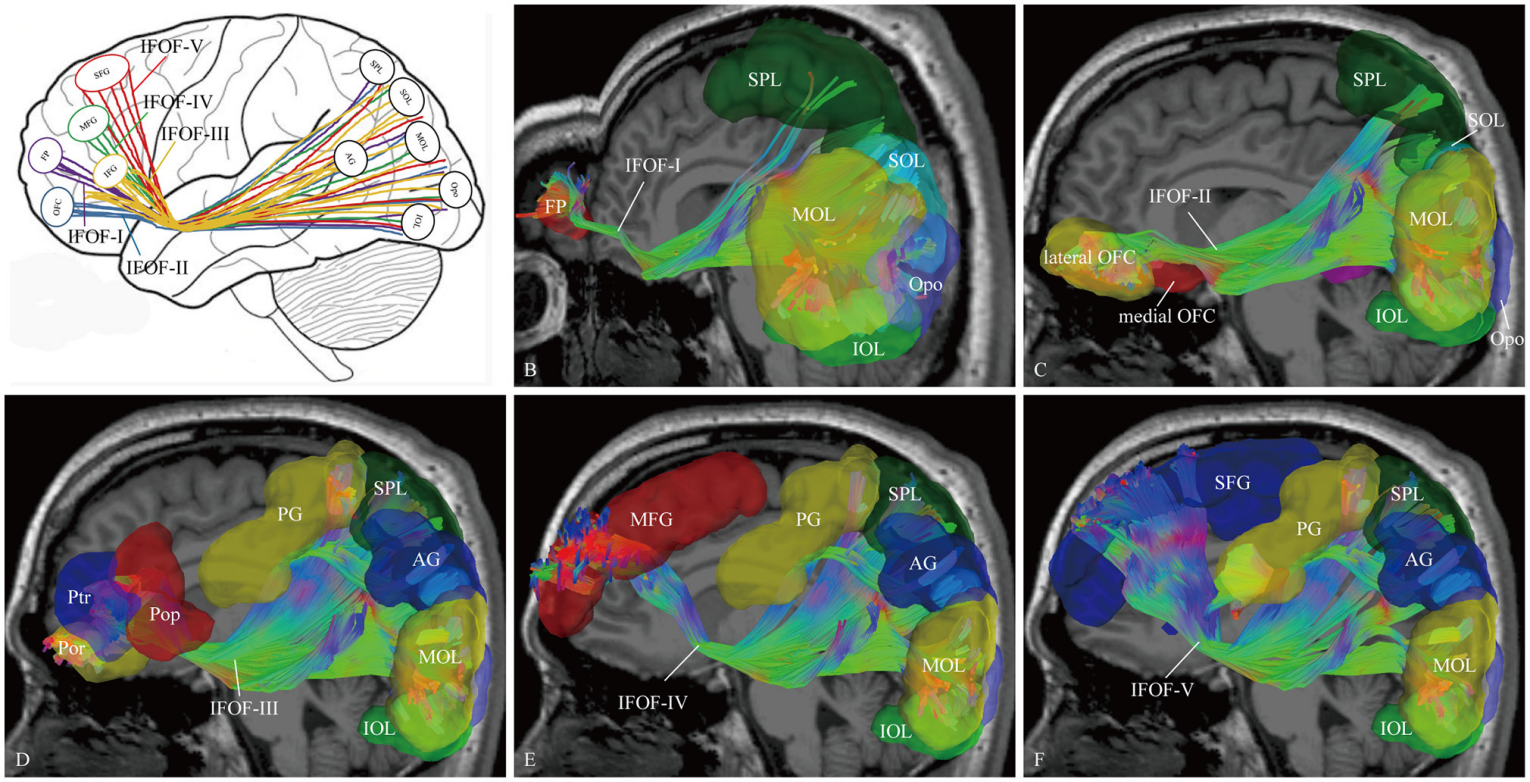
**The cortical endpoints of the IFOF and its five subcomponents (subject 1, left hemisphere). (A)** A diagram of the five subcomponents. **(B)** IFOF-I originated from the frontal pole cortex to the inferior occipital lobe, the occipital pole, the middle occipital lobe, the superior occipital lobe, and the superior parietal lobe. **(C)** IFOF-II originated from the orbito-frontal cortex to the occipital pole, the inferior occipital gyrus, the pericalcarine, the middle occipital lobe, the superior occipital lobe and the fusiform gyrus, and the superior parietal lobe. **(D)** IFOF-III originated from the inferior frontal gyrus (i.e., pars opercularis, pars triangularis, and pars orbitalis) to the inferior occipital gyrus, the middle occipital lobe, the occipital pole, the superior occipital lobe, the angular gyrus, the superior parietal lobe, and the postcentral gygus. **(E)** IFOF-IV originated from the middle frontal gyrus to the occipital pole, the inferior occipital gyrus, the middle occipital lobe, the superior occipital lobe, the angular gyrus, the superior parietal lobe, and the postcentral gygus. **(F)** IFOF-V originated from the superior frontal gyrus to the occipital pole, the inferior occipital gyrus, the middle occipital lobe, the superior occipital lobe, the superior parietal lobe, the angular gyrus, and the postcentral gygus. FP, frontal pole; OFC, orbito-frontal cortex; Pop, pars opercularis; Ptr, pars triangularis; Por, pars orbitalis; MFG, middle frontal gyrus; SFG, superior frontal gyrus; AG, angular gyrus; SPL, superior parietal lobule; PG, postcentral gygus; SOL, superior occipital lobe; MOL, middle occipital lobe; IOL, inferior occipital lobe; FG, fusiform gyrus; Opo, occipital pole.

### The spatial relationship of the IFOF with adjacent association tracts

**Figure 5:**
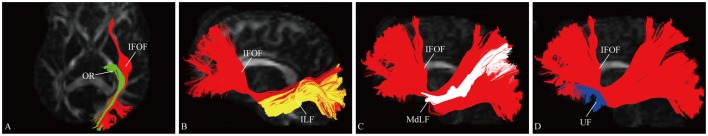
**Spatial relationship of the IFOF with adjacent association tracts. (A)** Axial slice showed the IFOF located superficially to the OR. **(B)** Sagittal view showed the IFOF located medially to the ILF. **(C)** Sagittal view showed the MdLF was immediately superficial to the fibers of the IFOF. **(D)** Sagittal view showed the IFOF was just above and medial to the UF at the level of the external/extreme capsule. OR, optic radiation; ILF, inferior longitudinal fascicles; MdLF, middle longitudinal fascicle; UF, uncinate fasciculus.

In order to visualize the spatial relationship of the IFOF with adjacent association tracts, the ILF, OR, MdLF, and UF were reconstructed (Figure 5).

At the roof of the temporal horn, the fibers of the IFOF are located medially to the ILF and superficially to the OR (Martino et al., 2010). The MdLF interconnects the superior temporal gyrus with the superior parietal lobe and parieto-occipital region, immediately superficial to the fibers of the IFOF (Wang et al., 2013). Based on the different orientation of the fibers of these three fascicles, they were clearly separated. The UF is a hook-shaped short-range tract connecting the frontal and temporal lobes (Leng et al., 2016). In previous DTI studies, the IFOF was often confused with the UF, as they are near each other in the region of the insula (Kier et al., 2004). According to our results, at the level of the external/extreme capsule, the IFOF narrows just above and medial to the UF.

### Quantitative study of the IFOF

The data of ILOF volume in 10 subjects were presented in Table 2. The relative volume of the IFOF in relation to the whole brain white matter volume was 18.5% (15.3–24.5%). The statistical results revealed no significant difference in the total volume of the IFOF between the left and right hemispheres (62.79 ± 19.77 ml vs. 54.43 ± 8.96 ml, *P* = 0.205). All originating and terminating distributions of the five subcomponents between males and females were listed in Tables 3-1, 3-2. There were no significant differences existed in distributions of the IFOF according to the sex, no matter in originating or terminating distributions (*P* > 0.05).

**Table 2:** **Volume studies of the IFOF in 10 individual subjects**.

**Volume(ml)**	**L**	**R**
1(male)	63.88	42.08
2(female)	84.75	66.67
3(female)	103.68	49.04
4(male)	64.89	63.32
5(female)	70.13	69.69
6(female)	40.72	49.58
7(male)	43.02	51.25
8(female)	44.91	52.61
9(female)	58.64	51.11
10(female)	53.26	48.90

*The IFOF relative volume in relation to the whole brain white matter volume was 18.5% (15.3–24.5%). No significant difference in the total volume of the IFOF between the left and right hemispheres (62.79 ± 19.77 ml vs. 54.43 ± 8.96 ml, P = 0.205). L, left hemisphere; R, right hemisphere*.

**Table 3-1:** **The originating distributions of the five subcomponents between males and females**.

**Variable**	**Total**	**FMG**	**MFG**	**MG**	**OFC**	**Pop**	**Por**	**Ptr**	**SFG**	**TFPG**	***P***
IFOF-L (M/F)	23/47	2/4	3/4	0/3	3/7	3/3	3/7	3/7	3/5	3/7	0.946
IFOF-R (M/F)	20/48	1/4	2/3	1/3	3/7	1/3	3/7	3/7	3/7	3/7	1.000
IFOF-I-L (M/F)	5/14	2/4	0/0	0/3	0/0	0/0	0/0	0/0	0/0	3/7	0.524
IFOF-I-R (M/F)	5/14	1/4	0/0	1/3	0/0	0/0	0/0	0/0	0/0	3/7	0.916
IFOF-II-L (M/F)	3/7	0/0	0/0	0/0	3/7	0/0	0/0	0/0	0/0	0/0	–
IFOF-II-R (M/F)	3/7	0/0	0/0	0/0	3/7	0/0	0/0	0/0	0/0	0/0	–
IFOF-III-L (M/F)	9/17	0/0	0/0	0/0	0/0	3/3	3/7	3/7	0/0	0/0	0.665
IFOF-III-R (M/F)	7/17	0/0	0/0	0/0	0/0	1/3	3/7	3/7	0/0	0/0	0.980
IFOF-IV-L (M/F)	3/4	0/0	3/4	0/0	0/0	0/0	0/0	0/0	0/0	0/0	–
IFOF-IV-R (M/F)	2/3	0/0	2/3	0/0	0/0	0/0	0/0	0/0	0/0	0/0	–
IFOF-V-L (M/F)	3/5	0/0	0/0	0/0	0/0	0/0	0/0	0/0	3/5	0/0	–
IFOF-V-R (M/F)	3/7	0/0	0/0	0/0	0/0	0/0	0/0	0/0	3/7	0/0	–

**Table 3-2:** **The terminating distributions of the five subcomponents between males and females**.

**Variable**	**Total**	**IOL**	**MOL**	**SOL**	**Opo**	**Pca**	**SPL**	**AG**	**FG**	**PG**	**Pre**	**ITG**	**MTG**	***P***
IFOF-L (M/F)	79/153	15/26	14/24	9/19	15/27	11/19	6/14	3/10	2/9	3/3	1/2	0/0	0/0	0.956
IFOF-R (M/F)	62/140	11/26	12/16	9/11	12/30	10/22	1/11	1/5	4/15	0/3	0/0	2/0	0/1	0.152
IFOF-I-L (M/F)	16/32	3/6	2/5	2/4	3/6	2/5	2/3	0/1	1/1	0/0	1/1	0/0	0/0	0.996
IFOF-I-R (M/F)	14/36	3/7	2/5	2/3	3/7	2/6	0/3	-	2/4	0/1	0/0	0/0	0/0	0.956
IFOF-II-L (M/F)	15/38	3/6	3/5	1/6	3/6	3/6	1/4	0/2	1/2	0/0	0/1	0/0	0/0	0.950
IFOF-II-R (M/F)	16/35	3/6	3/4	2/3	3/7	3/6	0/3	0/2	2/4	0/0	0/0	0/0	0/0	0.891
IFOF-III-L (M/F)	16/36	3/6	3/6	2/5	3/6	2/2	1/3	1/4	0/2	1/2	0/0	0/0	0/0	0.980
IFOF-III-R (M/F)	15/43	3/7	3/6	2/4	2/7	2/6	1/3	1/3	0/4	0/2	0/0	1/0	0/1	0.832
IFOF-IV-L (M/F)	16/20	3/4	3/4	2/1	3/4	2/3	1/1	1/1	0/2	1/0	0/0	0/0	0/0	0.894
IFOF-IV-R (M/F)	8/8	1/2	2/0	1/0	2/3	2/2	0/0	0/0	0/1	0/0	0/0	0/0	0/0	0.475
IFOF-V-L (M/F)	16/27	3/4	3/4	2/3	3/5	2/3	1/3	1/2	0/2	1/1	0/0	0/0	0/0	0.986
IFOF-V-R (M/F)	9/18	1/4	2/1	2/1	2/6	1/2	0/2	0/0	0/2	0/0	0/0	1/0	0/0	0.364

*Sexual originating and terminating distributions of the five subcomponents. There were no significant differences existed in distributions of the IFOF according to the sex, no matter in originating or terminating distributions (P> 0.05). M, male; F, female*.

## Discussion

In the present study, we investigated the trajectory, anatomical connectivity, and descriptive analysis of the IFOF in 10 subjects and a template of 90 subjects using high-angular-resolution fiber tractography. As for previous DTI reports, DSI approach demonstrated more higher resolution, and thus revealed a more complete connectivity pattern of the IFOF. Meanwhile, the subject-specific approach used in our study showed a greater inter-subjects variability, especially on the connection with the middle frontal gyrus, superior frontal gyrus, superior parietal lobe, angular gyrus, and postcentral gyrus. Furthermore, we also investigated whether there were discernible structural subdivisions in the IFOF based on white matter cortico-cortical connections. Five potentially different subcomponents were found within the IFOF, which could be achieved across 10 subjects and the template group.

Descriptions of the IFOF have a long history. In the last century, Curran described the IFOF for the first time as one of the major long-range association tracts integrating anatomically distant brain cortex. Over several decades, its anatomical existence has been frequently mentioned, but the results were controversial (Schmahmann and Pandya, 2007; Schmahmann et al., 2007). Recent advances in DTI technique, allowing “*in vivo*” dissection of the white matter, demonstrated the existence of IFOF in the human brain (Catani et al., 2002). However, tractography based on DTI failed to solve the problem of “kissing” and crossing fibers or to determine with accuracy the origin and termination of white matter tracts. It would produce false tracts and artifacts (Wedeen et al., 2008). We assumed that the long-range connection and the natural crossing of the IFOF with other tracts were the main roots of the biases in previous DTI results. In our study, we not only employed an advanced DSI scan involving a dense sampling of angular space for underlying water diffusion, but also reconstructed the tractography by GQI engaging a high-angular-resolution-based approach. They enabled us to acquire a significantly improved resolution to trace the fibers from one cortical region to another cortical or subcortical regions through complex crossings areas (Wedeen et al., 2008; Fernandez-Miranda, 2013). It provided detailed cortical site of the origin or termination of the IFOF without need for approximation (Wang et al., 2013; Fernandez-Miranda et al., 2014).

Previously, one ROI was widely used for the definition of the origin or termination of IFOF (Fernandez-Miranda et al., 2008a,b; Caverzasi et al., 2014). The manual ROI drawing was usually placed around the ventral part of the external capsule, which was somewhat subjective, and thus the fibers of IFOF could not avoid being influenced by the adjacent crossing tracts such as the UF and corona radiata. In our study, we reconstructed the IFOF based on the cortical ROIs defining by the Automated Anatomical Labeling. The anatomic subregions were automatically generated in light of individuality which were viewed as ideal for generating tracts (Desikan et al., 2006). Two ROI masks were used to select the fibers that only originated from the frontal lobe to the posterior regions. In our experience, the data acquired by this method were more accurate and more intuitive than that obtained by other methods (Wang et al., 2013; Fernandez-Miranda et al., 2014; Leng et al., 2016; Wu et al., 2016).

As the “gold standard”, post-mortem dissections have been widely employed to validate fiber tractography findings of the human white matter connective patterns. Over the past decades, although numerous microdissection studies have provided some basic anatomical information of the IFOF, a detailed analysis of the complex composition of this fiber bundle has not been reported. Only recently have two studies systematically investigated the anterior or the posterior cortical terminations of IFOF using post-mortem microdissection technique (Martino et al., 2010; Sarubbo et al., 2013). Now *in vivo*, we have not only reconstructed the results of these two studies in 10 healthy subjects and the template of 90 subjects, but also compared the connective difference among the subjects.

According to the anatomical dissection results, Martino et al. focused on the posterior terminations of IFOF, and firstly identified the posterior terminations both within and outside of the occipital lobes (Martino et al., 2010). However, they failed to identify the cortical terminations of IFOF within the frontal lobe. And they did not further confirm the results in the living human brain. Our results of *in vivo* tractography were consistent with their anatomical findings, well making up for the blanks. Moreover, we also found that there were some other terminations toward the angular gyrus, postcentral gyrus, and lingual gyrus. Indeed, the existence of the above cortex connections have been mentioned in some functional researches previously, which may be related to semantic processing (Hoeren et al., 2013; Moritz-Gasser et al., 2013; Willmes et al., 2014). This time, our results firstly provided importantly visible evidence to support the actual existence of these connections.

Recently, Sarubbo et al. has analyzed the frontal anatomical distribution of the IFOF by the post-mortem method, and attempted to confirm their findings using DTI (Sarubbo et al., 2013). They reported some new cortical terminations (i.e., middle frontal gyrus and superior frontal gyrus) in addition to the inferior frontal gyrus and orbito-frontal cortex. However, the study by Sarubbo et al. only involved the frontal connections, and not revealed the posterior components of the IFOF. Also, they performed DTI fiber tracking on just one left hemisphere. In accord with the anatomical findings of Sarubbo et al. our study identified more extended frontal connections beyond the previous “standard” IFOF. However, our results showed that there was a greater inter-subjects variability on the connections with the middle frontal gyrus and superior frontal gyrus, especially the fibers from the middle frontal gyrus, that we named as the IFOF-IV. This bundle of fibers was identified in 12 out of 20 hemispheres. And further investigation revealed that the above fiber tract was just a minor connectivity from the middle frontal gyrus to the posterior terminations in 8 out of 12 hemispheres. Similar results were obtained in the DSI template. Interesting, the inter-subjects variability was also revealed on the dorsal parieto-occipital connections among our 10 subjects. Indeed, this inter-subjects variability has been mentioned previously. For example, Martino et al. identified the superior parietal lobe connection of the IFOF in 9 out of the 14 hemispheres, while the posterior and basal temporal connections were showed in 8 out of the 14 hemispheres (Martino et al., 2010). From the above, we assumed that the individual variation might be the main root of previous controversies about the detailed anatomical definition of the IFOF.

To better understand the functional role of the white matter pathway, recent studies have revealed that the SLF and the AF are constituted by different subcomponents (Makris et al., 2005). In the same way, based on our tractography results, we proposed that the IFOF was not a single pathway, but rather was composed of distinct subcomponents of white matter at different levels. And thus we trend to define the IFOF as a “multi-function” bundle. Previously, Sarubbo et al. pointed out that there were two subdivisions within the IFOF (Sarubbo et al., 2013). Given that distinct characteristics of the frontal cortical areas might be ascribed to different functional roles, we suggested that the IFOF could be further segmented into five subcomponents.

### IFOF-I

The polar part of the frontal lobe (i.e., fronto-marginal gyrus and transverse fronto-polar gyrus, connected with the IFOF-I) is often designated as a single brain region, approximately corresponds to Brodmann's area (BA) 10 in the human brain. It is associated with many aspects of complex cognitive functions, such as social cognition, attention, multitasking, and episodic memory (Ramnani and Owen, 2004; Liu et al., 2013; Moayedi et al., 2015). These significant functions are primarily supported by white matter connectivity associating with the BA10 including the IFOF. Indeed, comparative morphological studies between human and non-human primate brains have showed that the human BA10 is proportionately much larger in absolute size. However, it is not disproportionately enlarged as comparisons with brain size (Semendeferi and Damasio, 2000). In addition, comparative analyses of the spatial organization of human BA10 cellular columns with BA10 in great apes, some significant differences have been identified between them, including cortical neuron organization. It means that the human BA10 contains more columnar interconnectivity (Semendeferi et al., 2011). In a recent study, Moayedi et al. used DTI to assess tractographic based dissections of the BA10 in 35 healthy subjects. They speculated that the white matter connections with the BA 10 (we named it as the IFOF-I) may play an important role in recent human cognitively special functions, which remained to be confirmed in further functional studies (Moayedi et al., 2015).

### IFOF-II

Obsessive compulsive disorder (OCD) is a chronically debilitating psychiatric disease, associating with considerable anxiety and socio-occupational dysfunction (Horwath and Weissman, 2000). Functional neuroimaging studies have shown that the cognitive abnormalities in OCD patients, such as insufficient cognitive-behavioral flexibility, executive function deficits and alteration in decision-making, are associated with white matter structures connecting with the frontal lobes (Saxena et al., 2001; Lawrence et al., 2009). A recent study by Garibotto and colleagues evaluated the patterns of directionality and organization of the major fiber bundles in 15 OCD patients. The significant changes were revealed in the anatomical connectivity of the orbito-frontal cortex of the frontal lobe with the parietal and occipital cortices along the IFOF-II (Garibotto et al., 2010).

### IFOF-III

For a long time, the Broca–Wernicke model of language implementation was widely adopt by generations of neuroscientists to solve language processing issues. The dorsal association via the SLF is the structural realization of these fiber connections (Poeppel and Hickok, 2004). Today, more studies have rediscovered a novel ventral stream connecting occipital, parietal, and posterior temporal regions to the Broca's areas as an independent second fiber connection of language network (Brauer et al., 2011, 2013). The fibers run through the extreme/external capsule confirming with our description on the IFOF-III. However, the distinctive roles of the dorsal and the ventral pathways playing in the language are still a matter of debate. Comparing with adults, more activities was found via the ventral fiber system for the language in children (Brauer et al., 2011). Recent studies on the neural connectivity of semantic memory has shown that a distributed left-lateralized association is consisted of the inferior frontal gyrus, the posterior lateral temporal region, the anterior temporal cortex, and the temporo-parietal junction (Nugiel et al., 2016). Therefore, Duffau et al. supported that the IFOF is the main structural pathway for semantic processing of the language (Duffau et al., 2013, 2014).

### IFOF-IV and IFOF-V

The superior frontal gyrus and the middle frontal gyrus components of the IFOF, as identified in our findings, are not always shown as parts of the IFOF in previous researches (Lawes et al., 2008; Voineskos et al., 2010; Vandermosten et al., 2012). Our frontal connection findings are in line with *ex vivo* fiber dissection results that can prove our data is not artificial (Sarubbo et al., 2013). This major discrepancy is probably based on different fiber-tracking resolution of our study. According to the fiber connections, Sarubbo et al. assumed that the IFOF-IV and the IFOF-V might take part in the semantic processing of language, visual conceptualization, and recognition, however, further clinical analysis are needed to confirm their hypothesis (Sarubbo et al., 2013).

There were several limitations in our study. First, only 10 subjects were traced using the subject-specific approach, which was still relatively inadequate. More subjects are required to provide a more detailed understanding of IFOF pathway variability in the human brain, and even to investigate the IFOF variability according to different gender and age. Second, our study only explored structural connectivity of IFOF, and further anatomical and functional investigations are needed to confirm our proposal related to subcomponents.

## Conclusions

The present study demonstrated the feasibility of high resolution diffusion tensor tractography with sufficient sensitivity to elucidate more anatomical details of the IFOF in the human brain. Our results were the first *in vivo* to present a more extended frontal and posterior connections beyond the previous 'standard' IFOF. In addition, according to our findings, we provided a novel framework for subdividing the IFOF, based both on its connectivity and function roles.

## Author contributions

Conceived and designed the experiments: YiW and YuW. Performed the experiments: YuW, YiW, and YoW. Data interpretation and picture preparation: YuW and DS. Contributed reagents/materials/analyses tools: YiW. Wrote the paper and revised the manuscript: YuW, DS, and YiW.

### Conflict of interest statement

The authors declare that the research was conducted in the absence of any commercial or financial relationships that could be construed as a potential conflict of interest.
